# Trends in patient age at planned oocyte cryopreservation

**DOI:** 10.1007/s10815-024-03237-z

**Published:** 2024-09-05

**Authors:** Tal Shavit, Joseph Hasson, Jordana Hadassah Hyman, Avi Tsafrir

**Affiliations:** 1 Faculty of Health Sciences, IVF Unit, Assuta Medical Centers, Ben-Gurion University, Be’er Sheva, Israel; 2 Faculty of Medicine, Hebrew University of Jerusalem, Jerusalem, Israel; 3 Department of Obstetrics and Gynecology, IVF Unit, Shaare Zedek Medical Center, Jerusalem, Israel

**Keywords:** Planned oocyte cryopreservation, Elective egg freezing, Egg banking, Age, Outcomes

## Abstract

**Purpose:**

The outcome of planned oocyte cryopreservation (POC) is inversely related to the age at the time of oocyte cryopreservation commencing in the mid-30 s. We sought to evaluate whether the age of women undergoing POC has changed over the last decade.

**Methods:**

The study employed a retrospective, observational multicenter design. It included all women who had at least one POC cycle in two large private IVF units belonging to the same medical organization in Israel. The main outcome measure was age at the first cycle. Data on the total number of women each year and their age at the first cycle were recorded.

**Results:**

Between 2011 and the end of 2023, 4488 women underwent POC. The average age at the first retrieval was 36.2 years (± 2.4). In 2011, the average age was 38.3 years (± 2.6), which decreased to 35.4 years (± 2.5) in 2023. The trendline indicates a decline in the average age of 3.0 months per year (*β* =  − 0.252, *F* = 301.8, *p* < 0.001). The proportion of women aged < 36 at their first POC cycle increased from 14% in 2011 to 54% in 2023.

**Conclusions:**

The age at the time of POC has significantly declined over the past decade. This trend may potentially lead to higher overall birth rates from POC, though further research is needed to confirm this hypothesis.

## Introduction

In developed nations, women’s age at first pregnancy increases consistently. Beginning in the mid-30 s, female fertility declines as age progresses, eventually leading to age-related infertility. The inevitable fertility decline with age is due to the reduced quantity and quality of oocytes. This decrease is caused by the aging processes of the human oocytes, resulting in lower chances to conceive and increased pregnancy loss rates. Current assisted reproductive technologies (ART) have limited success rates for women aged 40 and above [1]. After age 45, the chances to achieve a live birth with a woman’s own oocytes are negligible. Oocyte cryopreservation (OC) at a younger age may provide an opportunity to attain genetic motherhood later in life. Since the adoption of vitrification in IVF laboratories around 2000, the number of oocyte cryopreservation cycles has risen significantly [2]. Planned oocyte cryopreservation (POC) is the accepted term for oocyte vitrification aimed at addressing age-related fertility decline, previously known as “social freezing” [3]. Oocyte cryopreservation has been endorsed by both ESHRE [4] and ASRM [3]. The increase in oocyte cryopreservation over the past decade is primarily due to the rise in POC, rather than other indications such as prior to medical gonadotoxic treatments [5].

In a systematic review and meta-regression analysis on outcomes of POC including 8750 women, the mean age at time of POC was 37.2. Only 1517 (11.1%) of these women returned to use their oocytes, at mean age 41.8. The likelihood of achieving a live birth by using cryopreserved oocytes was 52% per woman who had POC at age 35 and younger, 34% when performed at age 36 to 39, and 19% for women at age 40 and older. However, while many clinicians advise women considering POC to complete the procedure at a younger age in order to achieve better chances for a live birth, only 19.4% of 8750 women who returned to use their oocytes had completed POC by age 35 [6].

Details on woman’s age at time of POC are seldom reported in the literature. According to SART data, the mean age of women who underwent cryopreservation declined from 36.7 in 2010 to 34.7 in 2016, while in Australia and New Zealand, the age profiles of those accessing oocyte cryopreservation remained mostly consistent. Unfortunately, the exact indication for oocyte cryopreservation was limited or absent in the American and Australian/New Zealand databases, respectively [2]. In a single-center study involving nearly one thousand women, the average age at first POC cycle decreased from 37.1 to 35.7 years [7].

In this study, we aimed to assess, within a large cohort over an extended period, whether the age at the time of POC has changed since this option became available and to determine the proportion of women who underwent POC before the age of 35.

## Materials and methods

Assuta is a medical organization that operates two IVF units located in central Israel. POC for women who wish to maintain better chances for live birth at advanced reproductive age was approved in Israel in 2011. Although fertility treatments addressing infertility and fertility preservation due to “medical” reasons (such as prior to gonadotoxic cancer treatments) are encompassed by national health insurance for all Israeli citizens, the cost of POC was covered privately beginning in 2011. This policy was updated in February 2022 to include planned oocyte cryopreservation (POC) in the national health insurance for women younger than 39 who are considered at risk for premature ovarian insufficiency, as defined by ovarian reserve test criteria.

For the purpose of this study, demographic data was extracted from the administrative database of the units regarding all women who underwent planned oocyte cryopreservation beginning from the first patient who had POC in 2011. Women who had cryopreservation for other indications such as unavailability of sperm on day of oocyte retrieval were not included in this study. Vitrification was used exclusively for oocyte cryopreservation in our units.

### Statistical analysis

For all ratio variables, means and standard deviations were calculated, and for all categorical.

Variables, numbers and percentages were calculated. In order to examine trends over time in patients age, the ANOVA test was performed.

Data were prepared in Microsoft Excel, and statistical analyses were conducted using the SPSS statistical software (version 21). The criterion for significance was alpha (*α*) = 0.05 (two-sided). Ethical approval was obtained from Assuta medical centers ethics committee, number ASMC 0064–23.

## Results

**Fig. 1:**
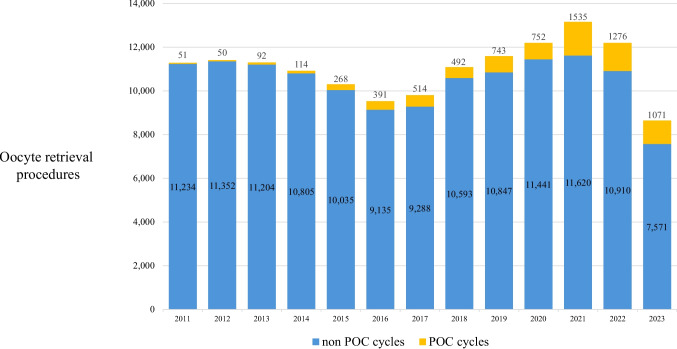
Annual POC and non-POC cycles. Proportion of POC cycles gradually increased from 0.5% of all cycles in 2011 to 12.4% in 2023. POC planned oocyte cryopreservation

Since 2011, when POC was first approved in Israel, until the end of 2023, a total of 4488 women underwent at least one cycle of POC in our units. The number of women who had POC per year increased gradually from 51 in 2011 to 939 in 2021 and declined in the two following years to 599 in 2023 (Fig. 1). In terms of retrieval cycles, POC cycles comprised 0.7% of oocyte retrieval procedures performed in both units in 2011. This gradually rose to 12.8% of retrievals in 2023.

**Fig. 2:**
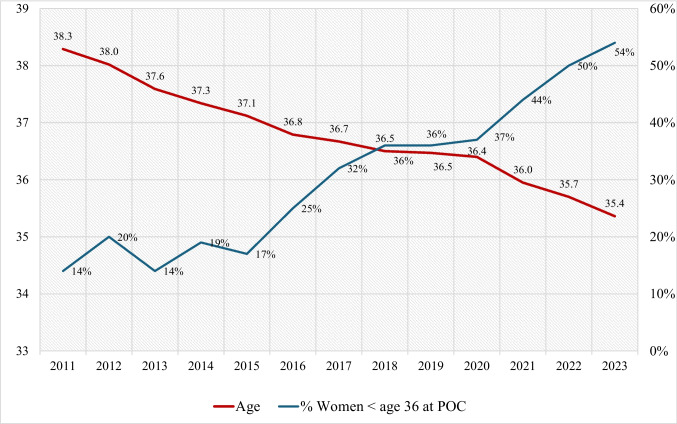
Mean age of women undergoing POC through 13 years. Mean age of women undergoing POC from 2011 to 2023 is represented by the red line (*n* = 4488). The blue line depicts the proportion of women undergoing POC before age 36 among all POC recipients. POC planned oocyte cryopreservation

The average age at first retrieval was 36.2 (± 2.4). The average age in 2011 was 38.3 (± 2.6) compared to 35.4 (± 2.5) in 2023. The trendline shows a decline in the average age of women over the period (2011–2023) of 3.0 months each year (*β* =  − 0.252, *F* = 301.1.8, *p* < 0.001). The proportion of women aged < 36 at time of first POC cycle increased gradually from 14% of all women who had POC in 2011 to 54% in 2023 (Fig. 2).

## Discussion

In this large cohort, we report that during the first decade of POC in our units, the mean age of women decreased significantly. Moreover, the proportion of women aged 35 years or younger who had POC comprised over 50% of all women having this procedure in 2023. This observation is not only statistically significant but is expected to yield meaningful clinical impact. In the largest single-center study so far on this topic, live birth rate per woman who had POC at age 35 and younger was 69% compared with 26% at an older age [8]. A recently published systematic review on outcomes of POC reported that live birth rates were 19, 34, and 52% per woman who had cryopreserved oocytes at age 40 and older, 36–39, and 35 or younger, respectively [6]. Therefore, according to our findings, improved live birth rates are expected for POC performed at a younger age, compared with the earlier years of this technology.

Our results are consistent with a single-center study from the USA including 1079 women. The authors observed a decline in mean age, from 37.1 to 35.7 from 2006 to 2020 [7]. While providing data on a longer time span compared to this study, the authors had a smaller cohort and did not report the relative proportion of younger women among all those who underwent POC.

We can only speculate on possible explanations for our observations. The first generation of POC was women at relatively advanced age who were at higher risk and probably more concerned about impaired fertility. POC may have seemed like an immediate solution, and therefore, older women rushed to cryopreserve their oocytes, while younger women responded in a slower manner as the option of POC became more widespread and less taboo.

Another explanation for our findings may be a positive result of public education and improved knowledge on fertility awareness including the effect of aging [9]. Success rates of fertility treatments are overestimated by the general public [10], by women attending fertility clinics [11], and even by experienced IVF patients [12, 13]. Women who had POC seem to have unrealistic expectations regarding this procedure as well [14–16], possibly encouraged by unbalanced information presented on clinics’ websites [17]. Not surprisingly, this might result in disappointment when dealing with actual outcomes. The key for improving POC outcome seems to be younger age. Numerous educational efforts emphasizing that POC is not a magical solution for age-related infertility and is less efficient if done at an advanced age seem to be effective according to our findings.

The SARS COVID-19 pandemic might have had an impact on women’s decision regarding POC as well, possibly by reflecting the importance of family ties and having children [18]. However, in a survey intended to evaluate the impact of the COVID-19 pandemic on women’s reproductive decision-making and specifically regarding interest in pursuing POC, only 15.2% believed that it influenced their likelihood of considering POC [19]. In another report, women who had POC in the UK were asked whether the COVID-19 pandemic made them more willing to freeze eggs. A similar proportion either agreed or disagreed with this statement [20]. While there were non-scientific press reports on an increasing number of women who had POC during the pandemic [21], we did not find data demonstrating an impact of the COVID-19 pandemic on the number of women choosing POC.

This study is one of the largest reporting on women who had oocyte cryopreservation specifically for the purpose of fertility preservation for anticipated age-related fertility decline. Previous reports, though much larger, could not provide data on the specific indication for oocyte cryopreservation [2]. Further, we collected all data starting from the first women who had POC in our units.

POC at a younger age enables improved fertility options for women; however, despite the benefits, there are also potential disadvantages to this trend that should be recognized and considered. According to present data, return rates of women who had POC are only 11% [6]. In the longest follow-up study to date, 38% of women returned after 10–15 years [22]. Return rates of women who had POC at younger age may even be lower. Possibly, younger women have better chances to engage in partnership for parenthood, which is the major factor in future return to use cryopreserved oocytes [15]. The accumulation of unused cryopreserved oocytes also presents a challenge for IVF clinics. It necessitates substantial resources for the long-term storage and maintenance of oocytes that might never be utilized.

Our study has several limitations. Since our study is based on administrative data, we could not report important details such as the number of oocytes cryopreserved, ovarian reserve test results, return rates, and live birth rates. Therefore, while we can hypothesize, we cannot provide direct evidence in this study that a decline in age at the time of POC will result in increased live birth rates. In addition, while the data set is large, this report reflects patient data from only two specific centers in central Israel. The general socio-economic status of residents in this area is higher than average compared to other parts of the country. Further, the cost of POC is significantly higher in our units compared with other units in Israel. This coincides with previous reports consistently showing that women who elect POC have higher income and education than the average population. These factors may limit the generalization of our findings.

## Conclusions

We report a continuous decline in the mean age of women who underwent POC over the past decade, with a rising proportion of these women being 35 years old or younger. Our study observed a significant trend in the reduction of age at the time of the first cycle of cryopreservation in a large cohort over a 13-year period. Specifically, in the final year of the study, more than half of the women who opted for POC were under 35 years of age. This trend is notable because it suggests that live birth rates are likely to improve compared with present outcomes. Further research is needed to repeat similar observations across different populations and healthcare systems and to follow up on the outcomes of women who underwent OC at younger ages to validate the predicted improvements.
